# Comparison of Protamine 1 to Protamine 2 mRNA Ratio and *YBX2* gene mRNA Content in Testicular Tissue of Fertile and Azoospermic Men 

**DOI:** 10.22074/ijfs.2015.4549

**Published:** 2015-10-31

**Authors:** Sahar Moghbelinejad, Reza Najafipour, Amir Samimi Hashjin

**Affiliations:** 1 Cellular and Molecular Research Centre, Qazvin University of Medical Sciences, Qazvin, Iran; 2 Department of Medical Genetics, Qazvin University of Medical Sciences, Qazvin, Iran

**Keywords:** Protamines, *YBX2*, *JHDM2A*, Azoospermia

## Abstract

**Background:**

Although aberrant protamine (*PRM*) ratios have been observed in infertile men, the mechanisms that implicit the uncoupling of *PRM1* and *PRM2* expression remain unclear. To uncover these mechanisms, in this observational study we have compared the *PRM1*/*PRM2* mRNA ratio and mRNA contents of two regulatory factors of these genes.

**Materials and Methods:**

In this experimental study, sampling was performed by a multi-step method from 50 non-obstructive azoospermic and 12 normal men. After RNA extraction and cDNA synthesis, real-time quantitative polymerase chain reaction (RT- QPCR) was used to analyze the *PRM1*, *PRM2*, Y box binding protein 2 (*YBX2*) and JmjC-containing histone demethylase 2a (*JHDM2A*) genes in testicular biopsies of the studied samples.

**Results:**

The *PRM1*/*PRM2* mRNA ratio differed significantly among studied groups, namely 0.21 ± 0.13 in azoospermic samples and -0.8 ± 0.22 in fertile samples. The amount of *PRM2* mRNA, significantly reduced in azoospermic patients. Azoospermic men exhibited significant under expression of *YBX2* gene compared to controls (P<0.001). mRNA content of this gene showed a positive correlation with *PRM* mRNA ratio (R=0.6, P=0.007). *JHDM2A* gene expression ratio did not show any significant difference between the studied groups (P=0.3). We also observed no correlation between *JHDM2A* mRNA content and the *PRM* mRNA ratio (R=0.2, P=0.3).

**Conclusion:**

We found significant correlation between the aberrant *PRM* ratio (*PRM2* under expression) and lower *YBX2* mRNA content in testicular biopsies of azoospermic men compared to controls, which suggested that downregulation of the *YBX2* gene might be involved in *PRM2* under expression. These molecules could be useful biomarkers for predicting male infertility.

## Introduction

Protamines (*PRMs*) comprise the largest amount of nucleoproteins in mature human sperm. These proteins are transcribed in steps 1-4 of spermatids (1) while synthesis of the corresponding proteins starts, with temporal delay, in step 4 spermatids (2). During spermiogenesis, *PRMs* replace somatic histones in a step-by-step manner, and cause higher DNA packaging in sperm compared to somatic cells. On the other hand, the condensed and insoluble nature of the sperm chromatin protects the genetic integrity of the parental genome during its transport through the male and female reproductive tracts (3).

Various studies reported abnormal expressions of *PRM* genes in sperm of infertile men. In addition, correlation of the altered *PRM1*/*PRM2* ratio has been shown with low sperm counts, decreased sperm motility and morphology, decreased fertilization ability and increased sperm chromatin damage (4-7). Several factors have been postulated and studied as possible causes of *PRM1*/*PRM2* deregulation (8-11). One of these candidate mechanisms is *PRM* gene polymorphisms that have also been reported for *PRM1, 2* genes. However most of these studies suggest that none of the *PRMs*’ single nucleotide polymorphisms (SNPs) and transition protein genes is likely to be a common cause of *PRM* abnormalities. Other factors that have attracted attention in this regard are transcription and translation regulatory genes of *PRM*. Several genes and proteins involved in *PRM1*/*PRM2* expression regulation have been identified and presented (12-16); until now, modification of these factors in infertile men with *PRM* deficiency attracted less attention. In this study we have proposed two *PRM* regulatory factors -Y box binding protein 2 (*YBX2*) gene and JmjC-containing histone demethylase 2a (*JHDM2A*) gene. These genes encode two important proteins involved in regulation of *PRM1*/ *PRM2* expressions (17, 18). *YBX2* is the human homologue of Xenopus DNA/RNA-binding and mouse MSY2 proteins (16), animal model studies show that this protein exists abundantly in testis tissue and is expressed in meiotic and post-meiotic germ cells (17). *YBX2* acts as an mRNA stabilizer and a transcription factor of *PRM* genes (18, 19). Consequently, *YBX2* loss of expression is likely to contribute to the nuclear condensation defects that occur in Msy2-null latestage spermatids (20). *JHDM2A* specifically regulates the expression of genes that encode transition protein 1 (Tnp1) and *PRM1*; this is necessary for proper chromatin reorganization during spermatid maturation by directly promoting transcription of TNP1 and *PRM1* genes (21). The current study analyzes the *PRMs* ratio in testicular tissue of azoospermic men. As *YBX2* and *JHDM2A* are involved in expression regulation of these genes, we have additionally evaluated whether *PRM* deficiency is related to downregulation of these genes.

## Materials and Methods

### Testicular tissue

This experimental study was approved by the Ethical Committee of the Faculty of Medical Sciences of Qazvin Medical Science University (Qazvin, Iran). After patients gave their informed written consent, testicular biopsies were obtained from 50 infertile men with a mean age of 31.3 ± 3.7 years; these patients were candidates for assisted reproductive technique (ART) and exhibited impaired spermatogenesis. In 12 patients with obstructive azoospermia after vasectomy, biopsies were performed out for diagnostic reasons during vasectomy reversal. These biopsies revealed normal spermatogenesis which served as controls; the mean age of these individuals was 35 ± 2.9 years. In this study patients were excluded if they had the following criteria: Y chromosome microdeletion, cystic fibrosis, varicocele, Klinefelter syndrome, or exposure to chemotherapy and radiation. In nonobstructive azoospermia patients, one part of the testicular tissue specimen was used for testicular sperm extraction, while the other part was cut into two pieces. One piece was immediately prepared and frozen for the RNA extraction procedure and the other piece was fixed in Bouin’s fixative, then embedded in paraffin.

### Histological evaluation

**Fig.1:**
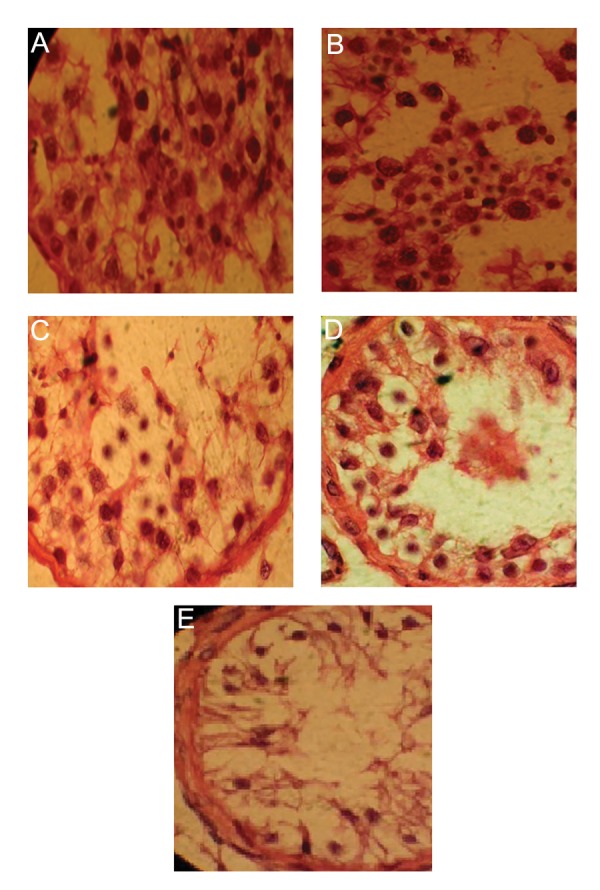
Results of hematoxylin and eosin staining of testis tissues. A. Hypospermatogenesis, B. Maturation arrest in round spermatid stage, C. Maturation arrest in spermatocyte stage, D. Maturation arrest in spermatogonial stage and E. Sertoli cell only. (magnification: ×1000).

We stained 5 μm paraffin sections in hematoxylin and eosin, and then scored the sections according to the modified Johnsen scoring system for histological evaluation (22). In this system of classification, all tubular sections in each piece of the testicular biopsy are evaluated systematically, and each is given a score from 1 to 10. Complete spermatogenesis with numerous spermatozoa is evaluated as score 10; slightly impaired spermatogenesis, numerous late spermatids, disorganized epithelium as score 9, less than five spermatozoa per tubule and few late spermatids as score 8, no spermatozoa, no late spermatids, and numerous early spermatids as score 7, no spermatozoa, no late spermatids, and few early spermatids as score 6; no spermatozoa or spermatids, and many spermatocytes as score 5, no spermatozoa or spermatids, and few spermatocytes as score 4; spermatogonia only as score 3, no germinal cells and Sertoli cells only as score 2; no seminiferous epithelium as score 1 (11). To follow this classification method, we divided the samples into two groups based on the above scoring: normal spermatogenesis (scores 9-10) and impaired spermatogenesis (scores 1-8) (Table 1, Fig .1A-E). It should be mentioned that our samples with score 8 had severe hypospermatogenesis.

**Table 1:** Characterization of patient candidates for ICSI


	Fertile men	Azoospermic men	P-value

Age (Y) (mean ± SD)	35 ± 2.9	31.3 ± 3.7	
Histology (score)	10	1-8	
Serum FSH (mIU/ml) (mean ± SD)	5.2 ± 2.3	14.26 ± 7.3	
ΔCt _(*PRM2*-*PRM1*)_,(mean ± SD)	-0.8 ± 0.22	0.21 ± 0.13	<0.0001
ΔCt _(*YBX2*-GAPDH)_,(mean ± SD)	3.3 ± 0.99	5.1 ± 1.2	<0.0001
ΔCt _(*JHDM2A*-GAPDH)_, (mean ± SD)	6.1 ± 1.1	6.3 ± 0.89	0.5


FSH; Follicle stimulating hormone and ICSI; Intracytoplasmic sperm injection.

### RNA extraction and first strand cDNA synthesis

After homogenizing frozen testis tissues using an Ultrasonic Processor UP100H (Hielsher, Germany), RNA was extracted with an RNeasy Mini Kit (Qiagene, Germany). The extracted RNA was frozen at -80˚C. We used a Nano Drop 2000c (Thermo, USA) to evaluate the quantity of isolated total RNAs. In this regard, RNA samples with A260/A280 ratios of >2 were selected for quantitative analysis. First strand complementary DNA (cDNA) synthesis was also performed using the Revert Aid First Strand cDNA Synthesis Kit (Thermo Scientific, Fermentas, Waltham, MA, USA).

### Real time quantitative polymerase chain reaction (RT-QPCR)

We designed four target genes primers and probes (*PRM1*, 2, *YBX2* and *JHDM2A*) using Gene Runner Softw are (Version 3.05, Table 2). A Taq Man RT- QPCR assay was carried out in final reaction volumes of 20 μl with 10 μl of Taq Man Master Mix (Takara, Shiga, Japan), 0.2 μM of forward and reverse primers, and 2 μl of cDNA. Thermal cycling was performed on the ABI-7500 (Applied Biosystems, Foster, CA, USA) sequence detection system by using the following cycling condition: 30 seconds at 95˚C as the first denaturation step, followed by 40 cycles at 95˚C for 5 seconds and 60˚C for 34 seconds. Each assay was repeated at least twice. The log-ratio of the transcript content in the samples was determined by the ΔCt method of relative quantification. The log-ratio of *PRM1* and *PRM2* was also calculated by ΔCt = Ct_*PRM2*_ – Ct_*PRM1*_, for *YBX2* and *JHDM2A*, these were ΔCt= Ct_*YBX2*_ – Ct_GAPDH_ and ΔCt= Ct_*JHDM2A*_ – Ct_GAPDH_. To study the correlation between *YBX2* and *JHDM2A* mRNA content and *PRM* log concentration, we used the calculating pattern of Steger et al. (6). Since Ct of *PRM1* did not change in the different groups, log-concentration of *YBX2* and *JHDM2A* were normalized to *PRM1* (ΔCt= Ct_*YBX2*_ – Ct_*PRM1*_ and ΔCt= Ct_*JHDM2A*_ – Ct_*PRM1*_) (23).

**Table 2:** Primer and probe sequences of target and internal control genes


Target and internal control genes	Sequence	Amplicon size (bp)

*PRM1*	F: TGACTCACAGCCCACAGAGT	124
	R: CTGCGACAGCATCTGTACCT	
	P:AGGCCAAGCCCATCCTGCAC	
*PRM2*	F:GCAAGAGCAAGGACACCAC	98
	R:GACACTGCTCTCGAAGGAGG	
	P: CGGAGCACGTCGAGGTCT	
*YBX2*	F:CCCTACCCAGTACCCTGCT	150
	R:CCTTCCTTCAACCCTTGATAA	
	P:CAGGAGGACCAAAGCAGCAGC	
*JHDM2A*	F: GTTCCACAAGCATTGACTGG	145
	R :CTGGTGCATTTGAAACATCC	
	P:TGCCAATCCTCCTGAACTGCAG	
*GAPDH*	F: TCAAGAAGGTGGTGAAGCAG	93
	R:CGCTGTTGAAGTCAGAGGAG	
	P: CCTCAAGGGCATCCTGGGCT	


*YBX2*; Y box binding protein 2, *JHDM2A*; JmjC-containing histone demethylase 2a, *PRM*; Protamin and GAPDH; Glyceraldehyde 3-phosphate dehydrogenase.

### Statistical analysis

In order to determine the significant differences between the studied groups, statistical analysis that included mean, standard deviation (SD), correlation coefficients (R^2^) and unpaired t test were performed with Prism (version 3) software. Additionally, linear correlations were tested using the Pearson coefficient of correlation. All tests were performed at a confidence level of 95%.

## Results

The mean Ct of *PRM1* in testis tissues were almost identical, 23.4 ± 3.6 (azoospermic) and 23.3 ± 1.5 (fertile). The mean Ct of *PRM2* in azoospermic men was 23.6 ± 1.8 and in the fertile group, it was 22.5 ± 0.41. Hence the expression ratio of *PRM2* was lower than the *PRM1* expression ratio in azoospermic men. The logarithm of the *PRM1*/*PRM2* mRNA ratio in azoospermic men was 0.21 ± 0.13 and for fertile men, it was -0.8 ± 0.22. This difference of ratios between fertile and azoospermic men was statistically significant (P<0.0001, Table 2).

In testicular tissues of azoospermic men with impaired spermatogenesis, the fold change of *YBX2* transcripts was 0.02 ± 0.019, and the log ratio of *YBX2* expression between azoospermic (5.1 ± 1.2) and fertile men (3.3 ± 0.99) significantly differed (P<0.0001, Table 2). In terms of *JHDM2A* gene expression, the differences of expression ratio were 6.1 ± 1.1 (fertile) and 6.3 ± 0.89 (azoospermic), which was not significant (P=0.5, Table 2).

**Fig.2:**
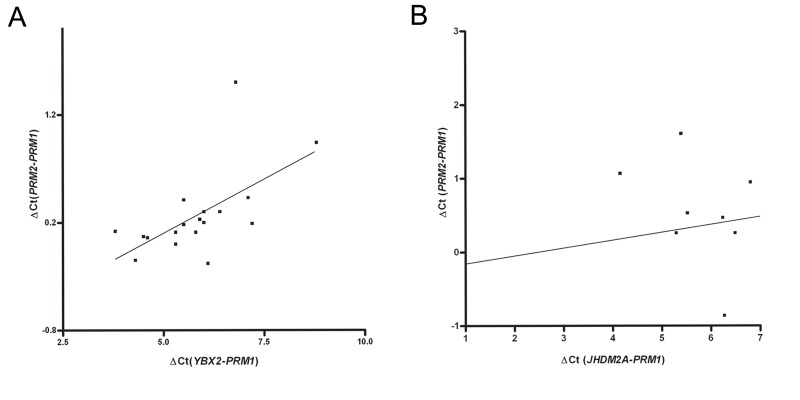
A. Correlation between the protamine-1 (*PRM1*) to *PRM2* log-ratio ΔCt (*PRM2*-*PRM1*) and normalized *YBX2* log-concentration ΔCt(*YBX2*-*PRM1*, R=0.6, P=0.007) and B. Correlation between the *PRM1* to *PRM2* log-ratio ΔCt (*PRM2*-*PRM1*) and normalized Jhdm2a log-concentration ΔCt (JH-*PRM1*, R=0.2, P=0.3).

The *YBX2* mRNA content revealed a positive linear correlation (R=0.6, P=0.007) with the *PRM1*/*PRM2* mRNA ratio or with *PRM2* deficiency (Fig .2A). However, we observed no linear correlation between *JHDM2A* mRNA content and the *PRM1*/*PRM2* mRNA ratio (R=0.2, P=0.3, Fig .2B).

## Discussion

In this research we observed a significant difference in *PRM1*/*PRM2* ratio between azoospermic and fertile men testicular biopsies (0.21 ± 0.13, 0.8 ± 0.22). *PRM2* changes were more than *PRM1*, which suggested different mRNA stabilities for the two molecules. A variety of studies reported a relationship between abnormal *PRM1*/ *PRM2* ratios and male infertility (4-6, 21, 23). On the basis of the studies which showed high *PRM1*/ *PRM2* ratios, it has been supposed that a reduction in *PRM2* expressions was responsible for aberrant *PRM1*/*PRM2* ratios in infertile males. Along this line, two studies reported complete selective absence of *PRM2* in infertile men (4, 24, 25). Lewis et al. (26) observed that in sperm of infertile men, *PRM2* downregulation occurred much more frequently than *PRM1* deregulation, because *PRM2* expression was more sensitive to the variation of regulatory controlling mechanisms than those for *PRM1*.

Our results confirmed the results from the above studies; we have observed significant downregulation of *PRM2* in the studied azoospermic men. *PRMs* genes are only transcribed in round spermatids and stored as silent mRNAs for later translation in elongating spermatids, in which transcription is no longer active. Since it is well justified to consider altered *PRMs* mRNA levels as a potential origin of altered protein levels (1, 11, 27), we have evaluated *PRM* expression at the mRNA level. Generally speaking, the mechanisms that underlie the uncoupling of *PRM1* and *PRM2* expression remain unclear, but generally four pathways in regulation of *PRM* gene expression have received more attention: the *PRM* genes themselves, transcription regulation, translation regulation, and downstream protein processing.

The *PRM1* and *PRM2* genes exist in a single chromatin domain in human sperm, and their transcription is regulated by the same upstream regulatory elements, thus making transcriptional and translational regulation a possible cause for aberrant *PRM1*/*PRM2* expression (28, 29). There are a number of regulatory proteins identified which are involved in repression or activation of *PRM* expression (29). In this regard, various animal models and *in vitro* studies have been performed, but scientists emphasize that future investigations should focus on aberrant expression, activation, and function of these regulatory factors in patients with deregulated *PRM1*/*PRM2* ratios (30, 31). To this end, we have focused on the expression ratio of two factors of *PRM* gene expressions.

*YBX2* is a transcription and translation regulatory factor, and a germ-cell-specific molecule essential for the production of functional spermatozoa. This gene is expressed in meiotic and post-meiotic germ cells, but its functional form is in round spermatids. Inactivation of *YBX2* can lead to male infertility. *YBX2*, also known as Contrin, is the human homologue of Xenopus DNA/RNA-binding and mouse MSY2 proteins (32, 33). To clarify the functional role of MSY2 in germ cells, Yang et al. (19) have generated Msy2-null mice. They found that mutant males had an abnormally high numbers of apoptotic meiotic spermatocytes, lacked spermatozoa in the epididymis, and were sterile. Their results emphasized the major role of this protein in male fertility (33). Hammoud et al. (34) investigated *YBX2* gene alterations in men with severe defects in spermatogenesis that included azoospermia, severe oligozoospermia, and *PRM* deregulation samples. Their results showed 15 polymorphic sites, of which 7 polymorphisms were present at a statistically higher frequency in patients with infertility, particularly in men with abnormal *PRM* expression. On the same path they showed that some SNPs in the *YBX2* gene occurred at a significantly higher incidence in men with *PRM* abnormalities than the control group. Our results, accordingly, showed significant downregulation of this gene in testicular tissues of azoospermic men compared to fertile men.

In terms of the molecular function of this protein, animal models and *in vitro* assay studies have shown that MSY2 acts as a transcription factor and an mRNA stabilizer which regulates expression of some testis specific genes at the transcription and translation levels, such as *PRM1, 2*. MSY2 marks specific mRNAs (those transcribed from Y-box promoters) in the nucleus for cytoplasmic storage, and thereby links mRNA transcription and storage/translational delay. In this process, MSY2 recognizes the CTG ATTGGC/TC/TAA sequence, a DNA motif in the promoter of numerous genes specifically expressed in male germ cells. After binding MSY2 to its consensus promoter sequence, it binds to transcripts of this gene, and stabilizes and represses their translation in cytoplasmic RNA-protein complexes (19, 35-38). Since *PRM1, 2* are regulated by the same upstream regulatory elements, we have expected significant under expression of *YBX2* to cause simultaneous downregulation of *PRM1, 2* in our azoospermic samples compared to fertile samples.

Unlike our expectation *PRM2* downregulated in the studied samples. We observed a positive linear correlation between downregulation of *PRM2* and *YBX2* genes. Statistically, R=0.6 exhibited an intermediate or good correlation, but this correlation was not perfect or 100%. This probably indicated that factors other than *YBX2* were involved in *PRM2* downregulation. Consistent with our results, one study has shown that decreased *PRM1* protein level is usually linked with posttranslational deregulation, but decreased *PRM2* is associated with low *PRM2* mRNA (30). *PRM2* transcripts are more susceptible to variations other than *PRM1* transcripts, downregulation of *YBX2* affects *PRM2* transcripts more than *PRM1* (26).

Another regulatory factor studied in this research was *JHDM2A*, also known as Jmjd1a or Kdm3a, was identified as an H3K9 demethylase (for monomethylation and dimethylation). *JHDM2A* was originally cloned as a testis-specific gene transcript. Results of immune histochemical analysis using anti- *JHDM2A* antibody showed an intense nuclear expression of this gene in round spermatids and a sub-nuclear distribution. Co-expression of *JHDM2A* gene with RNA polymerase II indicated that *JHDM2A* might contribute to transcriptional activation of some testis specific genes. *JHDM2A* has been shown to stimulate the transcriptional activation of transition nuclear protein1 and *PRM1* genes by bonding to the core promoter and removing H3K9 methylation (21). Histone demethylase *JHDM2A* is critical for Tnp1 and *PRM2* transcription and spermatogenesis. *JHDM2A* -deficient mice have infertility and smaller testes.

Despite the fact that animal model studies show the role of this protein in male infertility, we have not observed any significant difference in expression ratio of this gene between azoospermic and fertile men samples. Probably the role of this gene is not very influential in human spermatogenesis, and more samples must be studied. Regarding the correlation between this gene expression and *PRM2* downregulation our results have shown no positive linear correlation. Statistically, shows a weak or no correlation. Other studies have shown that this gene acts as a transcriptional activator of the *PRM1* gene. In addition, our samples displayed downregulation of the *PRM2* gene; therefore, expression of *JHDM2A* in our samples did not have an important role.

## Conclusion

We found significantly aberrant *PRM* mRNA ratios and a lower *YBX2* mRNA content in testicular spermatids of infertile men. In future studies the exact role of these molecules (*YBX2* and *JHDM2A*) and other *PRM* expression regulatory factors must be determined in human spermatogenesis.
